# Possible added value of thyroglobulin antibody (TgAb) testing in the evaluation of thyroidal status of subjects with overweight or obesity

**DOI:** 10.1007/s40618-022-01839-x

**Published:** 2022-07-04

**Authors:** P. Fierabracci, A. Basolo, G. Scartabelli, S. Bechi Genzano, G. Salvetti, G. Sotgia, M. Rotondi, L. Chiovato, G. Ceccarini, F. Santini

**Affiliations:** 1 Obesity and Lipodystrophy Center, Endocrinology Unit, University Hospital of Pisa, Via Paradisa 2, 56124 Pisa, Italy; 2 Consorzio Metis, University Hospital of Pisa, 56124 Pisa, PI Italy; 3 Unit of Internal Medicine and Endocrinology, Laboratory for Endocrine Disruptors, Istituti Clinici Scientifici Maugeri IRCCS, 27100 Pavia, PV Italy; 4 Department of Internal Medicine and Therapeutics, University of Pavia, Via S. Maugeri 4, 27100 Pavia, PV Italy

**Keywords:** Thyroglobulin autoantibodies, Hypothyroidism, Hyperthyrotropinemia, Obesity

## Abstract

**Purpose:**

An increase in serum TSH concentrations in the absence of thyroid disease, named isolated hyperthyrotropinemia, is frequently observed in subjects with obesity. It is directly associated with body mass index, and it is reversible following weight loss. Autoimmune hypothyroidism is frequently associated with obesity, it is usually progressive and needs replacement treatment with L-thyroxine. The aim of this study was to investigate the role of thyroglobulin antibodies (TgAb) to define the thyroidal status in subjects with overweight or obesity.

**Methods:**

This is a retrospective study including 749 consecutive adult patients with overweight or obesity. Of those, 76 were excluded from the analysis due to hyperthyroidism, previous thyroidectomy or radioiodine therapy for hyperthyroidism, hemiagenesis or drug-induced hypothyroidism. Serum thyrotropin (TSH), free thyroxine (FT4), free 3,5,3’-triiodothyronine (FT3), TgAb and thyroperoxidase antibodies (TPOAb) were measured in all patients.

**Results:**

Out of 673 patients, 408 did not have thyroid disease. Among patients with thyroid disease (*n* = 265), 130 had nodular disease with no humoral signs of thyroid autoimmunity and 135 (20%) had autoimmune thyroiditis, defined by the presence of TPOAb and/or TgAb. The prevalence of hyperthyrotropinemia, either directly measured or presumed based on L-thyroxine treatment at the time of data collection, was 63.9% in patients with both TgAb and TPOAb, 47.1% in those with isolated positivity of TPOAb, 42.8% in patients with isolated positivity of TgAb, and 14.5% in those with no detectable TgAb or TPOAb.

**Conclusions:**

Our results confirm a high prevalence of autoimmune thyroiditis (20%) in patients with obesity. TgAb may be associated with hypothyroidism in the absence of TPOAb. TgAb measurement may turn helpful to unravel a proportion of subjects that may have or may develop primary hypothyroidism requiring specific substitutive treatment.

**Supplementary Information:**

The online version contains supplementary material available at 10.1007/s40618-022-01839-x.

## Introduction

In past decades, the obesity prevalence has been constantly escalating in most countries, and by 2030 nearly one in two adults in the United States is projected to have obesity, and one in four to have severe obesity [1–4]. Obesity may be associated with hormonal alterations that are mainly secondary to excess body fat, and less frequently may be driven by primary endocrine diseases [5, 6]. Appropriate distinction between hormonal changes due to the excess of body fat and those depending on endocrine diseases that may be coexisting with obesity and contribute to weight gain (such as hypothyroidism or hypercortisolism) is mandatory for proper management of the patient. As far as thyroid function is concerned, patients with obesity often display an increase in serum thyrotropin (TSH) concentration, associated with low-normal free thyroxine (FT4) levels, in the absence of thyroid diseases [7–9]. This condition, named isolated hyperthyrotropinemia, may be interpreted as a compensatory mechanism aimed at counterbalancing the accelerated turnover of thyroid hormones caused by an increase in thyroid hormone disposal rate, which, in turn, activates the hypothalamus–pituitary–thyroid axis to maintain thyroid hormone concentrations within the normal range [10]. Recently, it has been shown that patients with obesity may display adipocyte and lymphocyte infiltration of the thyroid gland, not related to an autoimmune process, which could be associated with a reversible impairment of thyroid function, thus contributing to TSH elevation [11]. The European Society for Endocrinology (ESE) guideline on the endocrine work-up in obesity recommends that hyperthyrotropinemia (elevated TSH and normal FT4) should not be treated in obesity with the aim at reducing body weight  [6].

On the other hand, given that hypothyroidism is the most frequent endocrine disease in the general population [12], its association with obesity is a common finding. Indeed, based on a recent meta-analysis, the pooled prevalence of overt and subclinical hypothyroidism in obesity reaches 14.0 and 14.6%, respectively [5], although these numbers may actually overestimate the prevalence of the disease due to the higher proportion of female subjects in the published cohorts of patients with overweight or obesity. Based on these findings, the ESE guideline recommends that all patients with obesity are tested for thyroid function [6]. Testing for hypothyroidism should be based on TSH; if TSH is elevated, free T4 and thyroid peroxidase antibodies (TPOAb) should be measured. Guidelines also suggest that for the decision to treat or not to treat hyperthyrotropinemia, TSH level, thyroid antibodies, and age should be considered [6]. From a practical point of view, substitutive treatment with L-thyroxine (LT4) should be undertaken any time a cause for hypothyroidism and a foreseeable progression of thyroid failure are envisaged, particularly in specific clinical settings such as infancy or women of reproductive age [13]. Since current evidence is too weak to recommend testing of thyroglobulin antibodies (TgAb) in subjects with obesity, the ESE guideline suggests to consider it only in individual cases [6].

A similar strategy for the diagnosis of hypothyroidism in the general population is recommended by The European Thyroid Association (ETA) [14] and the American Thyroid Association (ATA) [15], both indicating measurement of TPOAb in patients with increased TSH, since the finding of elevated TPOAb allows to predict progression to overt hypothyroidism [14, 16]. However, although TPOAb has been identified as the most sensitive marker of autoimmune thyroiditis [17], evidence showed that TgAb may be predominant in specific clinical settings and may occur in the absence of TPOAb [17–23]. The current study was undertaken with the aim at understanding the potential role of TgAb measurement in the evaluation of the thyroidal status in subjects with overweight or obesity.

## Material and methods

This is a retrospective study including 749 consecutive patients (aged > 18 years) recruited at the Obesity and Lipodystrophy Center of the Endocrinology Unit, University Hospital of Pisa from January 2012 to February 2020. All patients had overweight or obesity according to body mass index (BMI) classification [24]. Of those, 76 were excluded from the analysis due to non-autoimmune acquired hypothyroidism (thyroidectomy *n* = 3, radioactive therapy *n* = 61, thyroid hemiagenesis *n* = 1, lithium-induced hypothyroidism *n* = 1) or hyperthyroidism (*n* = 10). Eventually, 673 patients were included in the analysis. A cohort of adult normal weight subjects with detectable TgAb and/or TPOAb (*n* = 404), who had access at the Endocrinology Unit during the year 2021, was retrospectively evaluated to calculate the proportion of subjects with isolated TgAb. All patients underwent measurement of body weight and height. Standing height, with no shoes, was measured (to the nearest 1 cm) using a stadiometer. Body weight, in light clothing, was measured (to the nearest 0.1 kg) with a digital electronic scale. BMI was calculated as the weight in kilograms divided by the square of the height in meters. Blood samples were drawn in the morning for the measurement of serum TSH, FT4, FT3, TgAb e TPOAb. The study was conducted in compliance with the principles of the Helsinki Declaration of 1975. Patients gave their consent for the use of clinical data for scientific purposes, and publication of this report was approved by the local Ethical Committee (CEAVNO—Comitato Etico Area Vasta Nord-Ovest).

FT4, FT3 and TSH were measured by an automated analyzer with a chemiluminescent method (Vitros 3600. Ortho-Clinical Diagnostics -Rochester, NY, USA); normal range was 0.7–1.7 ng/ml for FT4 and 2.7–5.7 ng/ml for FT3), 0.4–4.0 mU/L for TSH. TgAb and TPOAb were determined by an automated analyser enzyme immunoassay system (AIA-2000, Tosoh Corporation, Tokyo, Japan). The normal ranges of TgAb (< 30 UI/mL) and TPOAb (< 10 UI/mL) were established based on a local reference group. Thyroid ultrasonography was performed by a real-time instrument (Technos, Esaote Biomedica, Genova, Italy) with a 7.5 MHz linear transducer. The thyroid parenchima pattern was defined as hypoechoic or normoechoic by comparing the echo distribution of thyroidal parenchyma with that of neck muscles [25].

## Results

Demographic and anthropometric characteristics of the study population are shown in Table 1.

**Table 1:** Demographic and anthropometric characteristics of the study population

	Whole group *n* = 673	Women *n* = 491	Men *n* = 182
Age (years)	46.7 ± 14.0 (18–83)	45.8 ± 14.3 (18–83)	49.1 ± 12.9 (19–77)
Body weight (kg)	110.0 ± 25.2 (60–218)	103.0 ± 20.7 (6–-194)	128.7 ± 26.5 (72–218)
Height (m)	1.65 ± 0.09 (1.45–1.99)	1.62 ± 0.06 (1.45–1.83)	1.75 ± 0.08 (1.56–1.99)
BMI (kg/m^2^)	39.9 ± 7.9 (25.1–77.7)	39.2 ± 7.5 (25.1–77.7)	42.0 ± 8.6 (26.1–68.6)

Data are presented as mean ± SD (min–max)

**Fig. 1:**
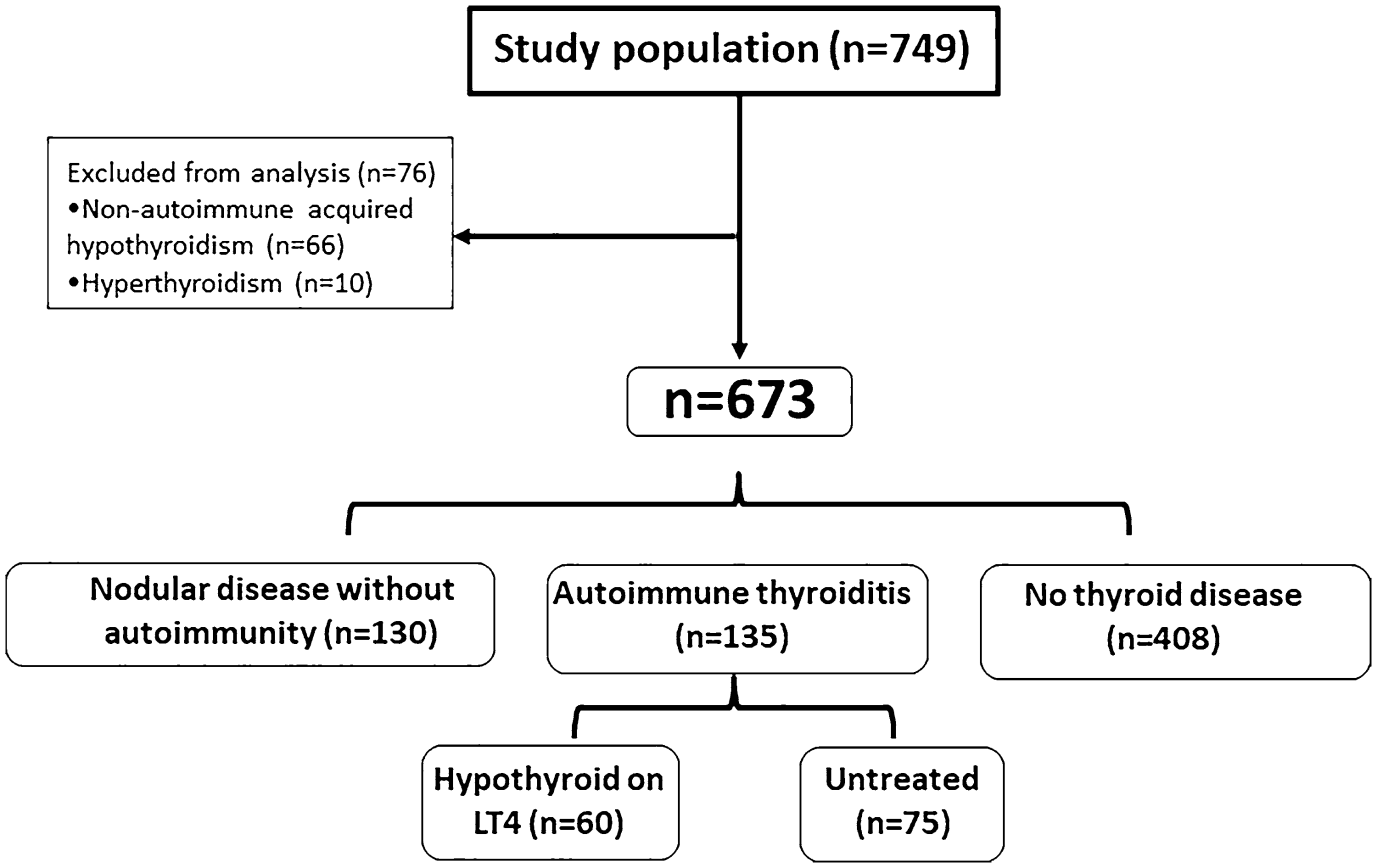
Study population based on the absence/presence of thyroid disease

Out of 673 patients, 408 did not have thyroid disease. Among patients with thyroid disease (*n* = 265), 130 had nodular disease with no humoral signs of thyroid autoimmunity and 135 (20%) had autoimmune thyroiditis, defined by the presence of TPOAb and/or TgAb (Fig. 1).

**Fig. 2:**
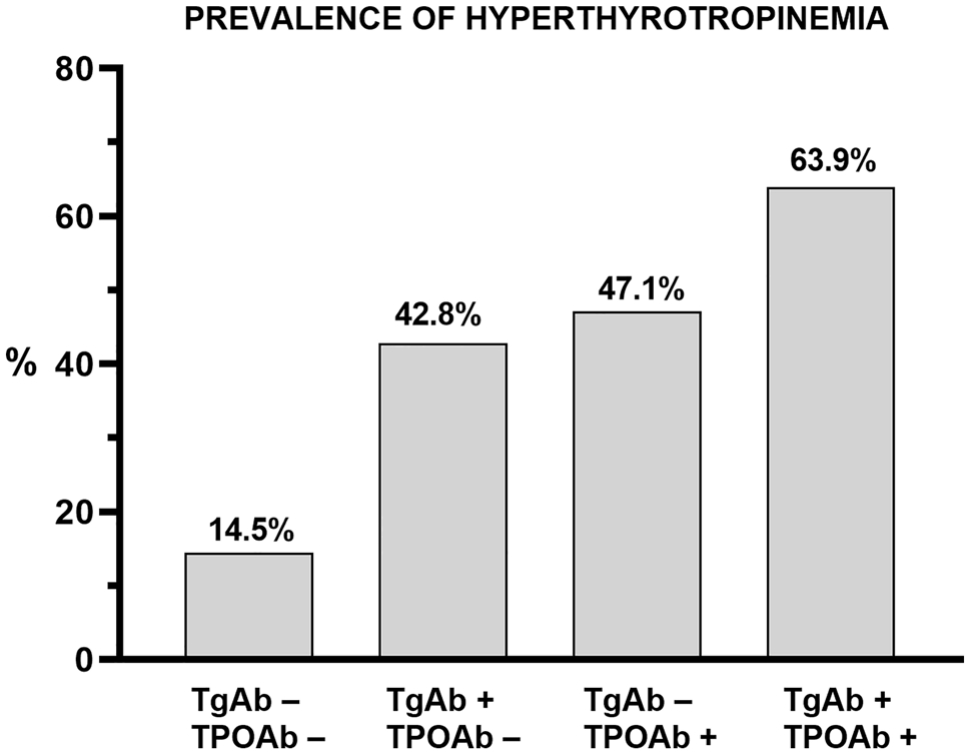
Prevalence of hyperthyrotropinemia in patients with or without thyroid antibodies

**Fig. 3:**
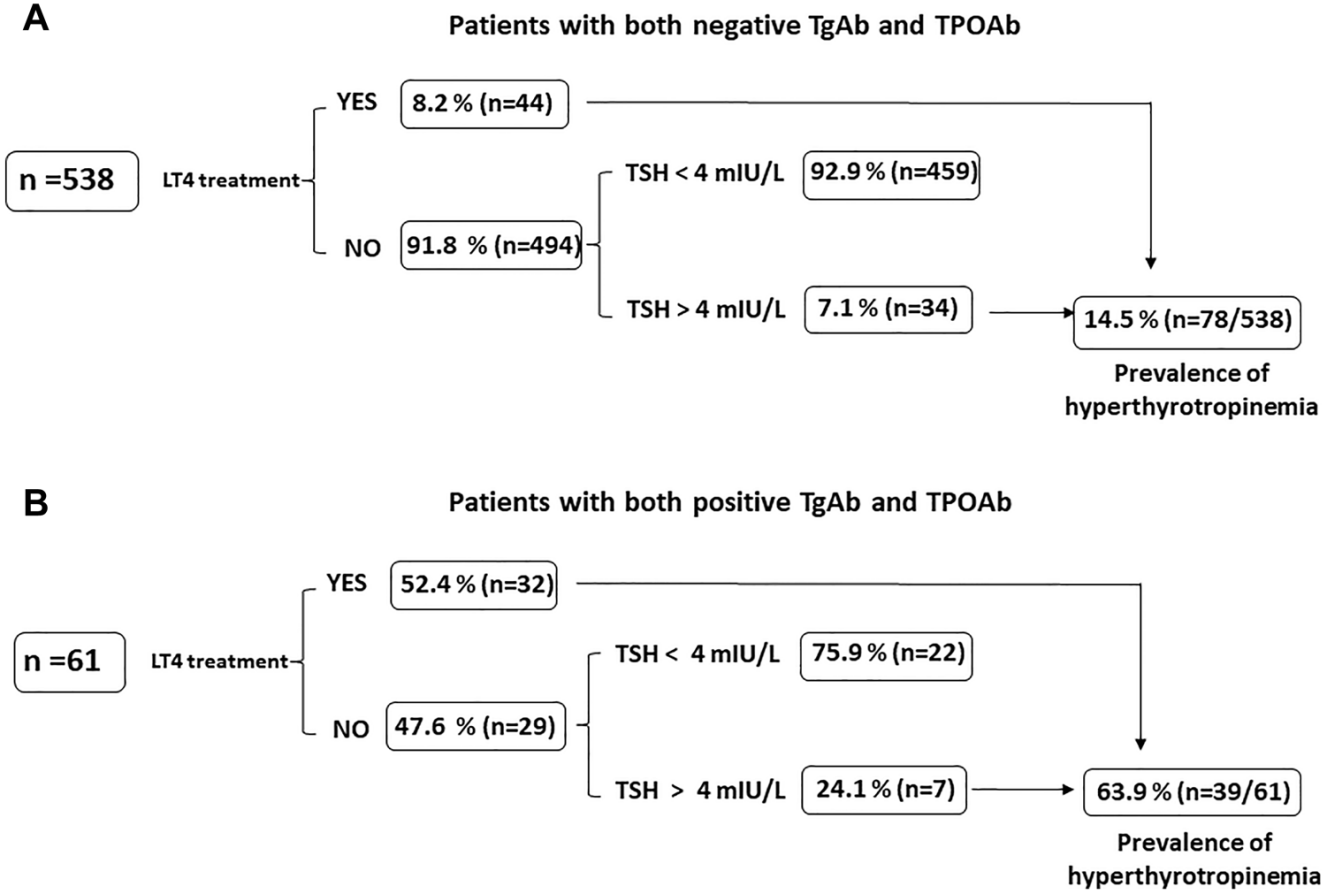
Panel **A** Prevalence of hyperthyrotropinemia in patients without thyroid antibodies; Panel **B** Prevalence of hyperthyrotropinemia in patients with both thyroperoxidase antibodies (TPOAb) and thyroglobulin antibodies (TgAb)

Among the 538 patients with no detectable TPOAb or TgAb, 44 patients were on LT4 treatment for previous detection of hyperthyrotropinemia (TSH > 4 mU/L). Among untreated patients, 34 had a TSH value above the normal range. Therefore, in the absence of thyroid antibodies, hyperthyrotropinemia was found in 78/538 (14.5%) patients (Figs. 2, 3A).

Concurrent detection of both TgAb and TPOAb occurred in 61/673 patients (9.1%). Of those, 32 were on LT4 treatment (52.4%). Among 29 untreated patients, 7 exhibited TSH concentrations above the normal range. Therefore, among patients with both TgAb and TPOAb, hyperthyrotropinemia was found in 39/61 (63.9%) patients (Figs. 2, 3B).

**Fig. 4:**
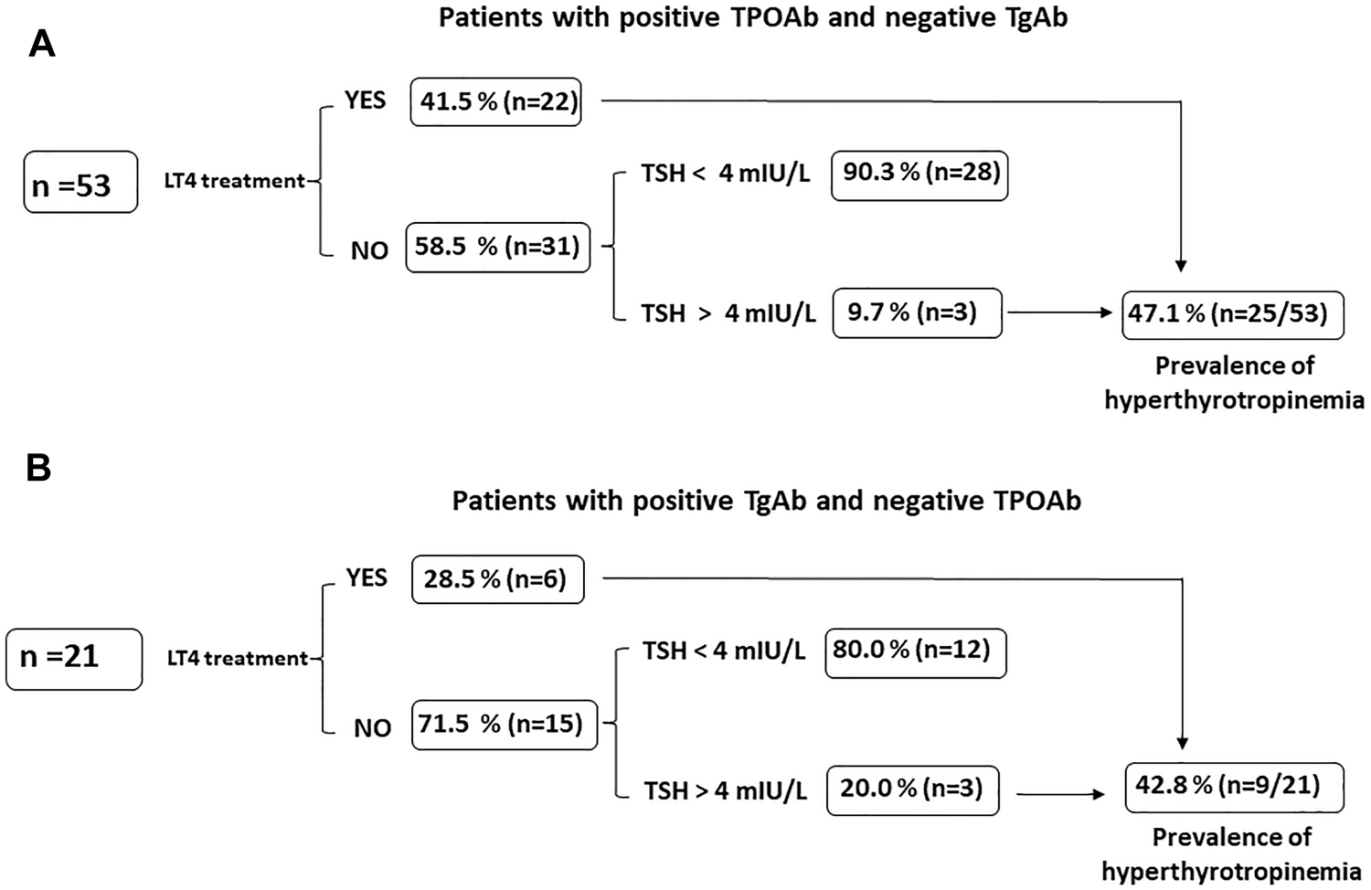
Panel **A** Prevalence of hyperthyrotropinemia in patients with isolated thyroperoxidase antibodies (TPOAb); Panel **B** Prevalence of hyperthyrotropinemia in patients with isolated thyroglobulin antibodies (TgAb)

Out of 673 patients, 53 had isolated positivity of TPOAb. Of those, 22 were on LT4 treatment (41.5%) whereas 31 were not (58.5%). In this latter group, three additional patients had TSH concentrations above the normal range. Therefore, among patients with isolated positivity of TPOAb, hyperthyrotropinemia was found in 25/53 patients (47.1%) (Figs. 2, 4A).

Out of 673 patients, 21 had isolated positivity of TgAb. Of those, six were on LT4 treatment (28.5%). Among untreated patients, three had TSH concentrations above the normal range. Therefore, among patients with isolated positivity of TgAb, hyperthyrotropinemia was found in 9/21 (42.8%) patients (Figs. 2, 4B).

**Fig. 5:**
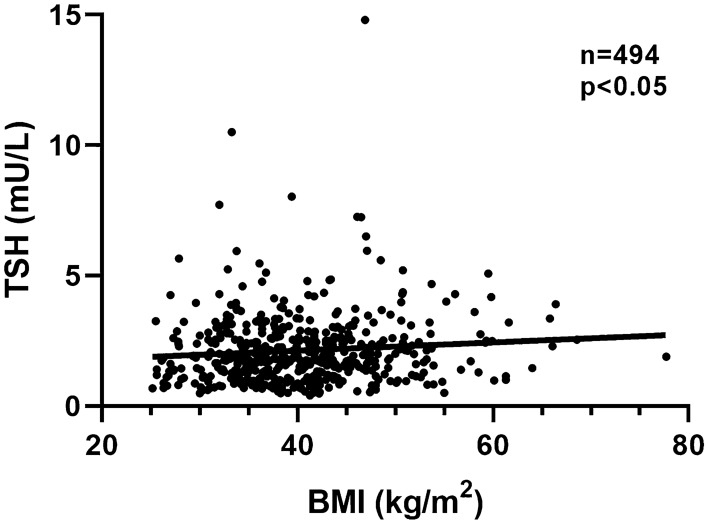
The graph shows the positive association (*r* = 0.09, *p* = 0.04) between serum TSH concentration and BMI in subjects with overweight or obesity with no detectable values of autoantibodies. Patients on LT4 treatment were excluded from the analysis

After exclusion of subjects on L-T4 treatment, the median TSH in subjects without thyroid antibodies (1.8 mUI/L) was significantly lower than that observed in subjects with autoimmune thyroiditis (2.4 mUI/L, *p* < 0.01). Furthermore, a significant positive association was observed between serum TSH concentrations and BMI values in patients with obesity or overweight and no thyroid autoantibodies (Fig. 5). No such a relationship could be demonstrated in subjects with autoimmune thyroiditis. In the cohort of normal weight subjects with detectable TgAb and/or TPOAb (*n* = 404), the prevalence of isolated positivity of TgAb was 25%, that of isolated positivity of TPOAb was 23%, and that of both TgAb and TPOAb positivity was 52% (vs. 16, 39 and 45%, respectively, in the overweight/obese group). Ultrasonographic evaluation of the thyroid gland was available in 108 patients with autoimmune thyroiditis. Of those 87 had a hypoechoic pattern and 21 showed a normoechoic pattern.

## Discussion

The increased prevalence of obesity in the last decades is associated with an increased demand of endocrine workout, aiming at identifying causes of obesity secondary to hormonal dysfunction, such as hypercortisolism and hypothyroidism, and a careful endocrinological evaluation of patients with obesity is necessary prior to any therapeutic approach [6]. The choice of the hormonal parameters to be tested is finalized at obtaining a timely evaluation with limited costs.

Chronic autoimmune thyroiditis is the most common cause of hypothyroidism in the adult population, with a prevalence of 10–12% [17, 26]. A higher prevalence has been reported in cohorts of subjects with obesity, which are biased by a higher female/male ratio [27–29]. In this retrospective study, we observed a prevalence of autoimmune thyroiditis of 20%, which was in line with that reported in previous cohorts of subjects with obesity. The prevalence of isolated positivity of TgAb in patients with obesity and humoral signs of autoimmune thyroiditis was slightly lower compared to that observed in lean controls and in previous studies [27, 28], although comparison is likely to be influenced by gender and age inhomogeneity among various study cohorts.

The assessment of thyroid autoantibodies is recommended to distinguish isolated hyperthyrotropinemia from autoimmune subclinical hypothyroidism, especially in patients with obesity, since thyroid autoantibodies may be considered a marker of the progression from subclinical to overt hypothyroidism [27, 30–33]. Interestingly enough, positive TgAb test has been found associated with symptoms burden in patients with Hashimoto’s thyroiditis [20]. Albeit subclinical hypothyroidism is not the primary cause of abnormal weight gain in patients with obesity [7–9], its treatment should be considered to improve concomitant cardiometabolic factors such as dyslipidemia [34], coronary artery disease [35] and depression [36], and to facilitate the adherence to therapeutic strategies aimed at achieving weight loss.

By assuming that patients on LT4 therapy were treated for hyperthyrotropinemia, our findings showed a 63.9% overall prevalence of hypothyroidism (overt or subclinical) in patients with both positive TgAb and TPOAb testing. Interestingly, the prevalence of hypothyroidism in patients with isolated positivity of TPOAb (47.1%) was only slightly higher than that observed in patients with isolated positivity of TgAb (42.8%), but definitely higher than the prevalence of isolated hyperthyrotropinemia (14.5%) in patients with no signs of thyroid autoimmunity. These results suggest that TgAb testing may be useful to distinguish autoimmune hypothyroidism from isolated hyperthyrotropinemia in patients with obesity. The latter condition, frequent in subject with obesity, is characterized by an increase in serum TSH concentration and low-normal FT4 levels, whereas FT3 concentrations might change in relation to the nutritional status [9, 37, 38].

Indeed, in the current study, we could confirm a positive association between serum TSH and BMI in subjects without humoral sign of thyroid autoimmunity not taking L-T4. In the absence of thyroid autoimmunity, increased TSH appears as a mechanism acting to counterbalance an accelerated turnover of thyroid hormones, which derives from an increase in thyroid hormone disposal rate, thus driving the response of the hypothalamus–pituitary–thyroid axis [9]. At variance, no association between BMI and serum TSH could be demonstrated in subjects with autoimmune thyroiditis suggesting that in such a context serum TSH levels depend mainly on the degree of thyroid dysfunction, as confirmed by significantly higher TSH values compared to subjects without thyroid autoimmunity.

 Whether isolated hyperthyrotropinemia may benefit from LT4 treatment has never been proven, and weight loss achieved with diet, pharmacological treatment or bariatric surgery can restore normal serum TSH concentrations [9]. On the other hand, demonstration of circulating thyroid antibodies is associated with progression from hyperthyrotropinemia to overt hypothyroidism, especially when there is an increase in TSH over time. By considering that LT4 treatment is highly effective, minimally expensive, and virtually devoid of side effects, it should be offered to these patients, also to facilitate the success of anti-obesity therapies. Based on the results of the current study, we think that TgAb testing could be cost effective in the diagnostic workout of obesity, to avoid missed diagnosis of autoimmune hypothyroidism and the consequent delay in the initiation of the substitutive hormonal treatment.

A hypoechoic pattern at neck ultrasound is a common feature of autoimmune thyroiditis, likely related to lymphocyte infiltration of the gland. However, in subjects with obesity, a hypoechoic pattern can be observed in the absence of autoimmune thyroiditis, thus reducing the diagnostic value of this marker [39].

We acknowledge that our study has several limitations. First, this is a retrospective, cross-sectional study that did not allow to monitor the course of the disease over time. Further, the prevalence of chronic autoimmune thyroiditis might be enriched due to the characteristics of the recruited individuals seeking treatment for obesity, predominantly composed by females who have higher risk of developing autoimmune thyroid disease. Third, there was no definite proof that all patients on LT4 therapy at the time of data collection were hypothyroid.

## Conclusion

In conclusion, our results confirm a high prevalence of autoimmune thyroiditis (20%) in patients with obesity. Furthermore, they indicate that TgAb may be associated with hypothyroidism in the absence of TPOAb. We believe that TgAb measurement should be considered to monitor thyroid function in patients with obesity, to avoid missing a proportion of subjects who may have or may develop primary hypothyroidism requiring specific substitutive treatment.

## Supplementary Information

Below is the link to the electronic supplementary material.

Supplementary file1 (DOCX 33 KB)
